# The role of macrophage scavenger receptor 1 (Msr1) in prion pathogenesis

**DOI:** 10.1007/s00109-021-02061-7

**Published:** 2021-03-23

**Authors:** Bei Li, Meiling Chen, Adriano Aguzzi, Caihong Zhu

**Affiliations:** 1 School of Basic Medical Sciences, Fudan University, Dong’An Road 130, Shanghai, 200032 China; 2 Institute of Neuropathology, University Hospital Zurich, Schmelzbergstrasse 12, CH-8091 Zurich, Switzerland

**Keywords:** Prion disease, Microglia, Macrophage scavenger receptor 1, Phagocytosis, Neuroinflammation

## Abstract

**Abstract:**

The progression of prion diseases is accompanied by the accumulation of prions in the brain. Ablation of microglia enhances prion accumulation and accelerates disease progression, suggesting that microglia play a neuroprotective role by clearing prions. However, the mechanisms underlying the phagocytosis and clearance of prion are largely unknown. The macrophage scavenger receptor 1 (Msr1) is an important phagocytic receptor expressed by microglia in the brain and is involved in the uptake and clearance of soluble amyloid-β. We therefore asked whether Msr1 might play a role in prion clearance and assessed the scavenger function of Msr1 in prion pathogenesis. We found that Msr1 expression was upregulated in prion-infected mouse brains. However, Msr1 deficiency did not change prion disease progression or lesion patterns. Prion deposition in Msr1 deficient mice was similar to their wild-type littermates. In addition, prion-induced neuroinflammation was not affected by Msr1 ablation. We conclude that Msr1 does not play a major role in prion pathogenesis.

**Key messages:**

- Msr1 expression is upregulated in prion-infected mouse brains at the terminal stage
- Msr1 deficiency does not affect prion disease progression
- Msr1 does not play a major role in prion clearance or prion pathogenesis
- Microglia-mediated phagocytosis and clearance of Aβ and prion may adopt distinct molecular pathways

**Supplementary Information:**

The online version contains supplementary material available at 10.1007/s00109-021-02061-7.

## Introduction

Prion diseases are transmissible and fatal neurodegenerative disorders that affect both human and animals. This disease category comprises Creutzfeldt-Jakob disease (CJD), fatal familial insomnia (FFI) and Gerstmann-Sträussler-Scheinker syndrome (GSS) in humans, scrapie in sheep and goat, bovine spongiform encephalopathy (BSE) in cattle, and chronic wasting disease (CWD) in cervids [1]. Prion diseases are thus far incurable. The infectious agent mainly consists of scrapie prion protein (PrP^Sc^), which is a misfolded isoform of the host-encoded cellular prion protein (PrP^C^). PrP^Sc^ acts as a propagon, which can seed a self-perpetuating reaction to recruit and convert PrP^C^ to an aggregated conformation. The deposition of PrP^Sc^ in the central nervous system (CNS), together with neuronal loss, spongiform changes (or termed vacuolization), astrogliosis and conspicuous microglial activation, constitutes the characteristic molecular pathology of prion disease [2].

Microglia are the primary innate immune cells and phagocytes of the CNS, exerting a neuroprotective role in prion pathogenesis [3]. We have reported that pharmacogenetic removal of microglia greatly enhances PrP^Sc^ accumulation in prion-infected cultured organotypic cerebellar slices (COCS) and in mice [4, 5]. However, the molecular mechanisms underlying prion clearance by microglia are largely unknown [1]. Lack of milk-fat globule EGF factor VIII (Mfge8) was reported to enhance prion pathogenesis in a mouse strain-dependent manner, suggesting that Mfge8 can facilitate engulfment of PrP^Sc^ aggregates by microglia [6, 7]. Developmental endothelial locuse1 (Del-1) is a structural and functional homolog of Mfge8 and therefore was a further candidate modifier of prion removal. However, Del-1 deficiency neither changed prion deposition nor prion pathogenesis in mice, suggesting that Del-1 does not complement Mfge8 in prion clearance [8]. Also triggering receptor expressed on myeloid cells 2 (TREM2), a phagocytic receptor expressed on microglia, modulates prion-induced microglial activation but does not contribute to prion clearance [9]. Hence, the molecules that are involved in prion clearance are complex and require further study.

Microglia express various receptors that facilitate sensing and phagocytosis of pathogens and misfolded protein aggregates; these include toll-like receptors (TLRs), scavenger receptors(SRs), Fc receptors, complement receptors, triggering receptor expressed on myeloid cells-2 (TREM2), myeloid cell surface antigen CD33, and others [10]. Importantly, variants of the *TREM2* and *CD33* genes are risk factors for Alzheimer’s disease (AD) [11–13], probably due to impaired uptake and clearance of amyloid-β (Aβ) [14, 15]. Although TREM2 is not a main transducer of prion clearance [9], the role of other microglial receptors in prion pathogenesis merits further investigations.

Msr1, also known as scavenger receptor a1 (Scara1), is a type II transmembrane glycoprotein mainly expressed by microglia in CNS [16]. Msr1 has been involved in many macrophage-associated physiological and pathological conditions such as neurodegenerative diseases [17]. As an important phagocytic receptor, Msr1 can mediate uptake of fibrillary amyloid β (Aβ) in vitro [18–20]. Msr1 deficiency in a mouse model of AD markedly accelerates Aβ accumulation and disease progression, whereas pharmacological upregulation of Msr1 leads to enhanced Aβ clearance. These results collectively suggest that Msr1 is essential for clearing soluble Aβ [21, 22]. Since both Aβ and prion are extracellular misfolded protein aggregates, they may share similar molecular pathways by which microglia take up and degrade the protein aggregates.

In this study, we aimed to investigate whether Msr1 may have a similar function in prion clearance and pathogenesis. We first determined the Msr1 expression in a mouse model of prion disease and found Msr1 expression was significantly increased in prion-infected mouse brain. After prion inoculation, we then observed that *Msr1*^-/-^ mice showed disease progression similar to their hemizygous (*Msr1*^+/-^) and wild-type (*Msr1*^+/+^) littermates. Besides, prion deposition and seeding dose were not altered by Msr1 deficiency, suggesting that Msr1 is not involved in prion clearance. Furthermore, Msr1 deficiency did not affect prion-induced neuroinflammation. We therefor conclude that Msr1 is not a major player in prion clearance and does not influence prion pathogenesis.

## Material and methods

### Ethical statement

All animal experiments were carried out in strict accordance with the Rules and Regulations for the Protection of Animal Rights (Tierschutzgesetz and Tierschutzverordnung) of the Swiss Bundesamt für Lebensmittelsicherheit und Veterinärwesen and were pre-emptively approved by the Animal Welfare Committee of the Canton of Zürich (permit # 41/2012).

### Animals

*Msr1*^-/-^ mice were generated by inserting a neomycin cassette into the EcoRI site in exon 4, which encodes the alpha helical coiled-coil structure essential for the formation of functional trimeric receptors ([23]; JAX stock #006096). *Msr1*^-/-^ mice were first backcrossed to C57BL/6 J mice to obtain *Msr1*^+/-^ offspring, which were then intercrossed to generate *Msr1*^+/+^ (wild type), *Msr1*^+/-^ and *Msr1*^-/-^ mice for experiments described here. All animals were maintained in high hygienic grade facility under a 12 h light/12 h dark cycle (from 7 am to 7 pm) at 21±1 °C and fed with diet and water ad libitum.

### Prion inoculation

Mice were intracerebrally (i.c) inoculated with 30 μl of brain homogenate diluted in PBS with 5% BSA and containing 3 × 10^5^ LD50 units of the Rocky Mountain Laboratories scrapie strain (passage 6, thus called RML6). Mice were monitored and actions were taken to minimize animal suffering and distress according to details described previously [24]. Scrapie was diagnosed according to clinical criteria (ataxia, limb weakness, front leg paresis and rolling). Mice were sacrificed by CO_2_ inhalation on the day of appearance of terminal clinical signs of scrapie (specific criteria referred to [24]), organs were taken and then were either snap-frozen for biochemical analysis or fixed in 4% formalin for histological assessment. The time elapsed from prion inoculation to the terminal stage of disease was defined as incubation time for the survival study.

### Quantitative real-time PCR (qRT-PCR)

Total RNA from was extracted using TRIzol (Invitrogen Life Technologies) according to the manufacturer’s instruction. The quality of RNA was analyzed by Bioanalyzer 2100 (Agilent Technologies), RNAs with RIN > 8 were used for cDNA synthesis. cDNAs were synthesized from ~ 1 μg total RNA using QuantiTect Reverse Transcription kit (QIAGEN) according to the manufacturer’s instruction. Quantitative real-time PCR (qRT-PCR) was performed using the SYBR Green PCR Master Mix (Roche) on a ViiA7 Real-Time PCR system (Applied Biosystems). The following primer pairs were used: GAPDH sense 5´-CCA CCC CAG CAA GGA GAC T-3´; antisense, 5´-GAA ATT GTG AGG GAG ATG CT-3´. Msr1 sense 5´- TGA ACG AGA GGA TGC TGA CTG -3´; antisense, 5´- GGA GGG GCC ATT TTT AGT GC -3´. TNFα sense, 5´-CAT CTT CTC AAA ATT CGA GTG ACA A-3´; antisense, 5´-TGG GAG TAG ACA AGG TAC AAC CC-3´. IL-1β sense, 5´-CAA CCA ACA AGT GAT ATT CTC CAT G-3´; antisense, 5´-GAT CCA CAC TCT CCA GCT GCA-3´. IL-6 sense, 5´-TCC AAT GCT CTC CTA ACA GAT AAG-3´; antisense, 5´-CAA GAT GAA TTG GAT GGT CTT G -3´. Expression levels were normalized using GAPDH.

### Immunohistochemistry

Prion-infected brain tissues were harvested and fixed in formalin, followed by treatment with concentrated formic acid for 60 min to inactivate prion infectivity and embedded in paraffin. Paraffin sections (2 μm) of brains were stained with hematoxylin/eosin (HE) to visualize prion-induced lesions and vacuolation. For the histological detection of partially proteinase K-resistant prion protein deposition, deparaffinized sections were pretreated with formaldehyde for 30 min and 98% formic acid for 6 min, and then washed in distilled water for 30 min. Sections were incubated in Ventana buffer and stains were performed on a NEXEX immunohistochemistry robot (Ventana instruments, Switzerland) using an IVIEW DAB Detection Kit (Ventana). After incubation with protease 1 (Ventana) for 16 min, sections were incubated with anti-PrP SAF-84 (1:200, SPI bio, A03208) for 32 min. Sections were counterstained with hematoxylin. To detect astrogliosis and microglial activation, brain sections were deparaffinized through graded alcohols, anti-GFAP antibody (1:300; DAKO, Carpinteria, CA) were applied for astrogliosis, and anti-AIF1 antibody (1:1000; Wako Chemicals GmbH, Germany) was used for highlighting activated microglial cells. Stainings were visualized using DAB (Sigma-Aldrich), and hematoxylin counterstain was subsequently applied. Sections were imaged using a Zeiss Axiophot light microscope. Quantification of SAF84 staining was performed on acquired images. Regions of interest were drawn, and the average signal density was quantified using Image J software (National Institutes of Health).

### Western blot analysis

To detect PrP^C^ in the mouse brains, one hemisphere of each brain was homogenized with RIPA buffer. Total protein concentration was determined using the bicinchoninic acid assay (Pierce). ~8 μg proteins were loaded and separated on a 12% Bis-Tris polyacrylamide gel (NuPAGE, Invitrogen) and then blotted onto a nitrocellulose membrane. Membranes were blocked with 5% wt/vol Topblock (LuBioScience) in PBS supplemented with 0.1% Tween 20 (vol/vol) and incubated with primary antibodies POM1 in 1% Topblock (400 ng ml^−1^) overnight. After washing, the membranes were then incubated with secondary antibody horseradish peroxidase (HRP)-conjugated goat anti–mouse IgG (1:10,000, Jackson ImmunoResearch, 115-035-003). Blots were developed using Luminata Crescendo Western HRP substrate (Millipore) and visualized using the Stella system (model 3000, Bio-Rad). To avoid variation in loading, the same blots were stripped and incubated with anti-actin antibody (1:10,000, Millipore). The PrP^C^ signals were normalized to actin as a loading control.

To detect PrP^Sc^, prion infected forebrains were homogenized in sterile 0.32 M sucrose in PBS. Total protein concentration was determined using the bicinchoninic acid assay (Pierce). Samples were adjusted to 20 μg protein in 20 μl and digested with 25 μg ml^−1^ proteinase K for 30 min at 37 °C. PK digestion was stopped by adding loading buffer (Invitrogen) and boiling samples at 95 °C for 5 min. Proteins were then separated on a 12% Bis-Tris polyacrylamide gel (NuPAGE, Invitrogen) and blotted onto a nitrocellulose membrane. POM1 and horseradish peroxidase (HRP)-conjugated goat anti–mouse IgG were used as primary and secondary antibodies, respectively. Blots were developed using Luminata Crescendo Western HRP substrate (Millipore) and visualized using the FUJIFILM LAS-3000 system. To detect GFAP and AIF1 in prion-infected brains by Western blot, 20 μg of total brain protein were loaded and anti-GFAP antibody (D1F4Q) XP Rabbit mAb (1:3000; Cell Signaling Technology, 12389), anti-AIF1 antibody (1:1000; Wako Chemicals GmbH, Germany, 019-19741) and horseradish peroxidase (HRP)-conjugated goat anti-rabbit IgG (1:10,000, Jackson ImmunoResearch, 111-035-045) were used as primary and secondary antibodies, respectively. Actin was used as the loading control.

### Real-time quaking induced conversion assay (RT-QuIC)

RT-QuIC assays of prion-infected mouse brain homogenates were performed as previously described [8, 25]. Briefly, recombinant hamster full-length (23–231) PrP was expressed in Rosetta2(DE3)pLysS *E. coli* competent cells and purified by affinity chromatography using Ni2^+^-nitrilotriacetic acid Superflow resin (QIAGEN). In the RT-QuIC assay, recombinant HaPrP was used as substrate for PrP^Sc^-catalyzed conversion. RT-QuIC reactions containing HaPrP substrate protein at a final concentration of 0.1 mg mL^-1^ in PBS (pH 7.4), 170 mM NaCl, 10 μM EDTA, and 10 μM Thioflavin T were seeded with 2 μL of serially diluted brain homogenates in a total reaction volume of 100 μL. NBH- and RML6-treated brain homogenates were used as negative and positive controls, respectively. The RT-QuIC reactions were amplified at 42 °C for 100 h with intermittent shaking cycles of 90 s shaking at 900 rpm in double orbital mode and 30 s resting using a FLUOstar Omega microplate reader (BMG Labtech). Aggregate formation was followed by measuring the thioflavin T fluorescence every 15 min (450 nm excitation, 480 nm emission; bottom read mode).

### Statistical analysis

Results are presented as the mean of replicas ± standard error of the mean (SEM). Statistical significance between experimental groups was assessed using an unpaired Student’s *t* test. For prion inoculation experiments, incubation times were analyzed using the Kaplan-Meier method and compared between groups using Log-rank (Mantel-Cox) test. *p* values < 0.05 were considered statistically significant.

## Results

### Prion infection upregulates Msr1 expression in the mouse brain

**Fig. 1:**
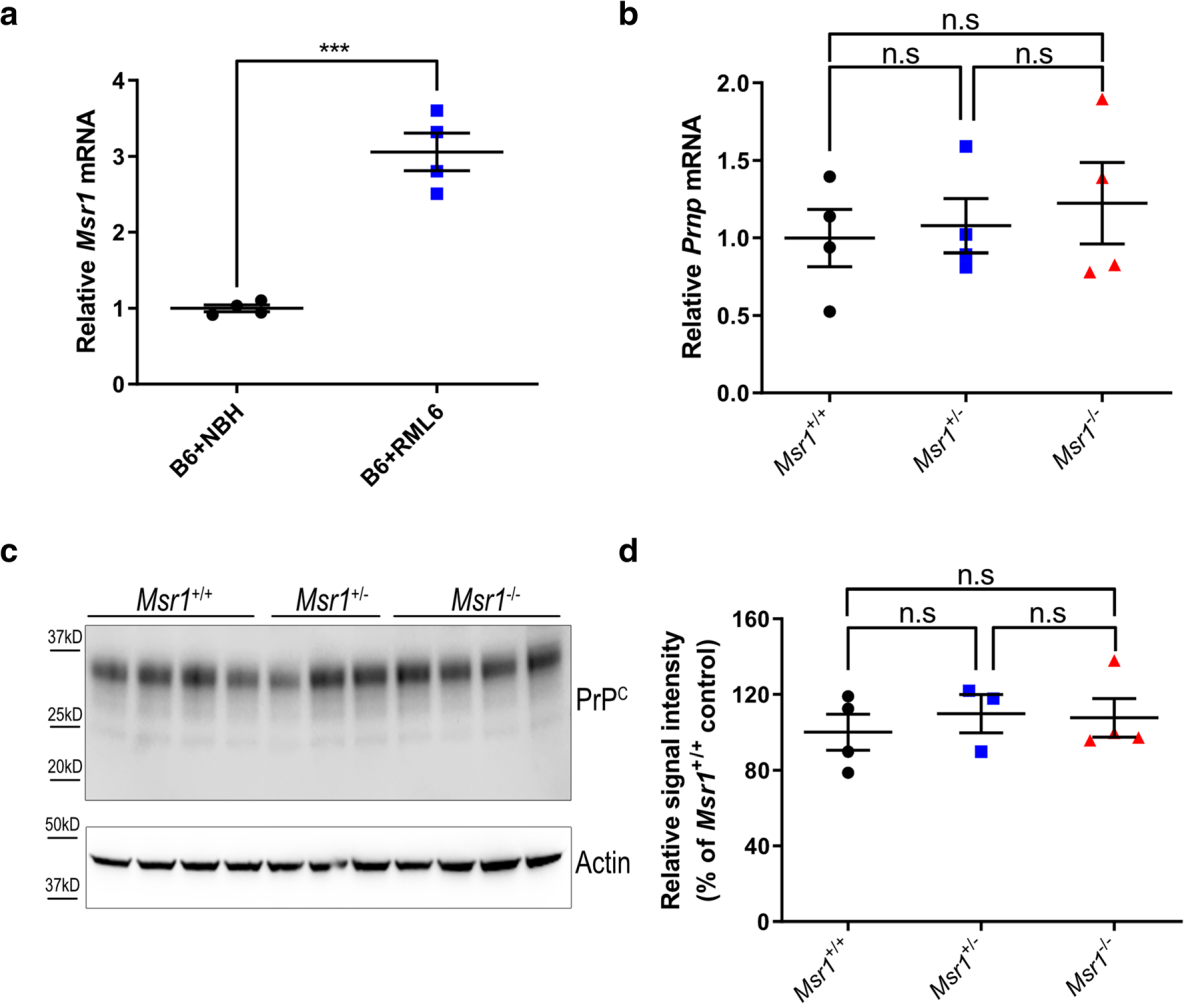
(**a**) qRT-PCR of *Msr1* expression in mouse brains. *Msr1* expression was significantly increased in RML inoculated mouse brain, compared to NBH inoculated mouse brain (*n* = 4, *p* < 0.001). (**b**) *Prnp* qRT-PCR in *Msr1*^+/+^ (WT), *Msr1*^+/-^, and *Msr1*^-/-^ mouse brains (*n* = 4, n.s *p* > 0.05). No significant difference of *Prnp* mRNA was observed between three groups. **c** and **d** PrP^C^ Western blot (**c**) and densitometric quantification of the Western blot (**d**) in *Msr1*^+/+^ (WT), *Msr1*^+/-^, and *Msr1*^-/-^ mouse brains (n=3~4, n.s p>0.05). No significant difference of PrP^C^ protein was observed between three groups. Abbreviations: *Msr1*, macrophage scavenger receptor 1 gene; *Prnp*, murine prion gene; PrP^C^, cellular prion protein; qRT-PCR, quantitative real-time polymerase chain reaction; RML6, Rocky Mountain Laboratories scrapie strain, passage 6; NBH, noninfectious brain homogenates; WT, wild type

Expression of *Msr1* could be upregulated by various forms of brain injury and neurodegeneration [16, 26–29]. To test whether prion infection affects *Msr1* expression in the mouse brain, we intracerebrally (i.c) inoculated 30 μl of diluted CD1 mouse brain homogenate containing 3 × 10^5^ LD50 (50% lethal dose) units of RML6 (a prion strain originated from the Rocky Mountain Laboratory, serially passaged to No. 6, hence termed RML6) into C57BL/6 J mice. C57BL/6 J littermates inoculated with noninfectious brain homogenate (NBH) were used as control. Prion-inoculated mice were euthanized and brains were collected when they showed severe scrapie sign and reached the terminal stage of disease, NBH-inoculated control mice were sacrificed after the same incubation time. Since we could not commercially obtain a sensitive and specific antibody detecting the Msr1 protein in mouse brains (supplemental Fig. 1), we assessed *Msr1* expression levels using quantitative reverse-transcription PCR (qRT-PCR). The results showed that *Msr1* expression was significantly enhanced in prion-inoculated mouse brain, compared to NBH inoculated mouse (Fig. 1a), suggesting that *Msr1* expression can be efficiently upregulated by prion infection.

Consistent with our qRT-PCR results, a time course study of hippocampal transcriptome of prion-infected mice by RNA sequencing (RNA-Seq) showed that *Msr1* mRNA level was significantly increased at the terminal stage of RML-inoculated mice, but not in NBH-inoculated control mice (supplemental Fig. 2a). Interestingly, however, during the preclinical stage and even at the early stage after onset of clinical symptoms, *Msr1* mRNA levels were not significantly changed (supplemental Fig. 2a). Considering that Msr1 is mainly expressed by microglia in the CNS, and the microglia can be conspicuously activated and proliferate upon prion infection, we aimed to determine whether the upregulation of *Msr1* mRNA is due to the increased microglia number. We found that mRNA levels of various microglia markers including *Aif1, CD68*, *and Itgax* were almost simultaneously induced in prion-inoculated mice from 16 weeks post-inoculation on (supplemental Figs. 2b–d), when *Msr1* mRNA was not significantly altered yet [30]. Moreover, a cell-type-specific ribosomal profiling using CX3CR1-Cre^ER^ mice revealed that Msr1 was indeed upregulated in microglia only at the terminal stage (supplemental Figure 2E) [31]. Therefore, these results suggest that upregulation of *Msr1* expression was not merely an effect of increased microglia number after prion infection, but displayed unique pattern and dynamics.

PrP^C^ expression levels in mouse brains are the major determinants of the susceptibility to prion diseases and progression rate of the diseases [32, 33]. To assess whether Msr1 deficiency could influence PrP^C^ expression, we performed qRT-PCR and Western blot to measure *Prnp* mRNA transcripts and PrP^C^ protein levels in *Msr1*^+/+^, *Msr1*^+/-^, and *Msr1*^-/-^ littermates. The results showed that Msr1 deficiency altered neither the *Prnp* mRNA (Fig. 1b) nor the protein levels of PrP^C^ (Figs. 1c–d, uncropped Western blots are shown in Supplementary Fig. 3a–b) in mouse brains.

### Prion disease progression, lesion pattern, or PrP^Sc^ accumulation are not altered by Msr1 deficiency

**Fig. 2:**
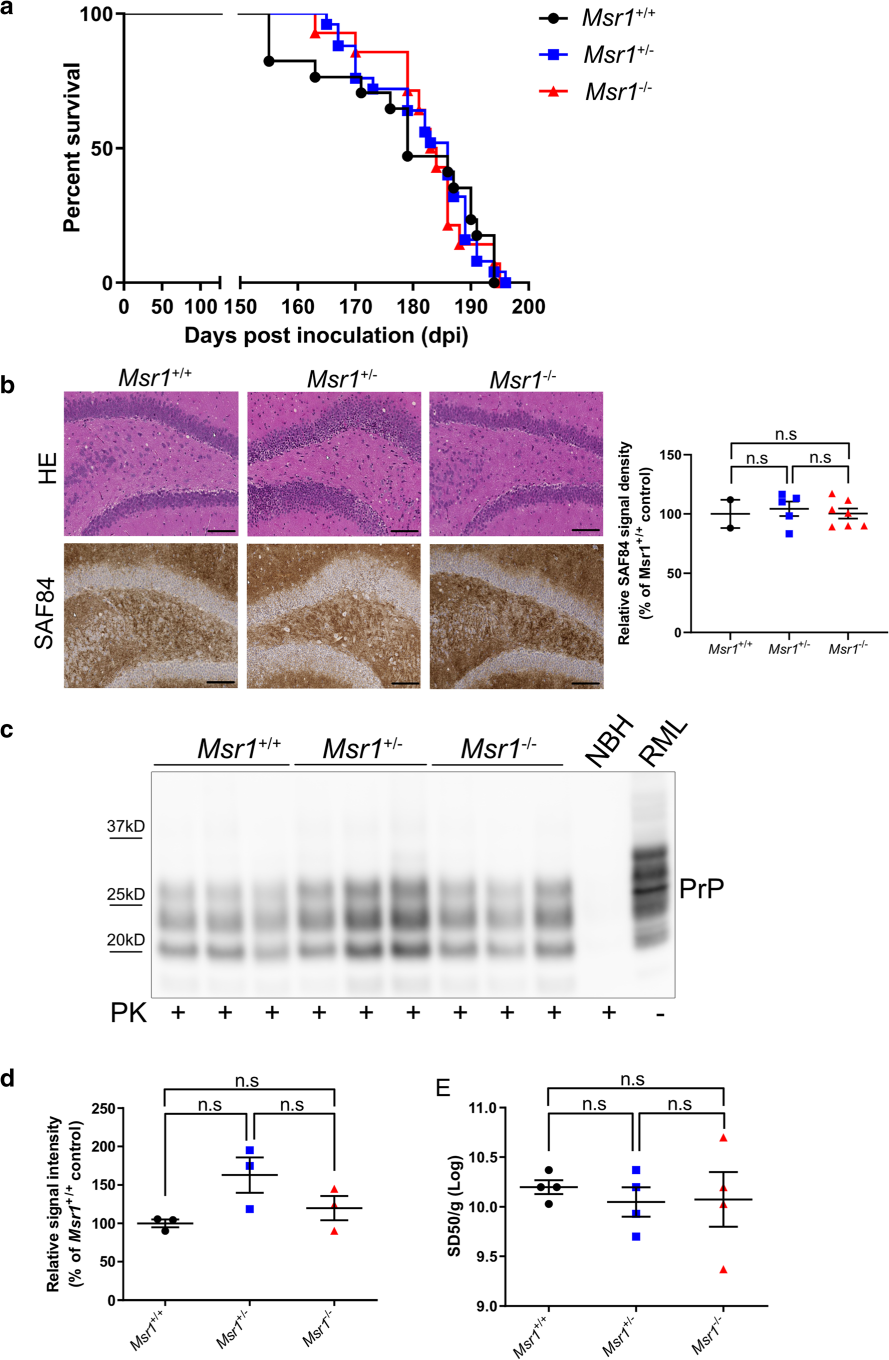
(**a**) Survival curve of *Msr1*^+/+^ (WT), *Msr1*^+/-^ and *Msr1*^-/-^ littermates intracerebrally inoculated with RML6. There was no significant difference between three groups in both genders (*n* = 14–25, n.s *p* > 0.05). (**b**) Left: Representative histology of terminally sick mouse brains from *Msr1*^+/+^ (WT), *Msr1*^+/-^ and *Msr1*^-/-^ littermates stained for H&E and SAF84. Right: quantification of SAF84 staining. There was no obvious difference between three groups in vacuolation, lesion pattern and PrP^Sc^ deposition in hippocampus (*n* = 2–7, n.s *p* > 0.05). **c** and **d** Western blot for proteinase K resistant PrP^Sc^ in terminally sick mouse brains (**c**) and densitometric quantification of the Western blot (**d**). There was no significantly difference between *Msr1*^+/+^ (WT), *Msr1*^+/-^ and *Msr1*^-/-^ littermates (*n*=3, n.s *p* > 0.05). (E) RT-QuIC assay of terminally sick mouse brains from *Msr1*^+/+^ (WT), *Msr1*^+/-^ and *Msr1*^-/-^ littermates showed similar level of 50% prion seeding dose in these three groups (*n* = 4, n.s *p* > 0.05). Abbreviations: Msr1, macrophage scavenger receptor 1; PrP^Sc^, scrapie-associated prion protein; RML6, Rocky Mountain Laboratories scrapie strain, passage 6; NBH, noninfectious brain homogenates; WT, wild type; RT-QuIC, real-time quaking-induced conversion assay

To assess the function of Msr1 in prion pathogenesis, we next tested whether Msr1 ablation could affect prion disease progression and alter prion-mediated lesion pattern in mouse brains. We intracerebrally (i.c) inoculated 30 μl of RML6 prions into *Msr1*^+/+^, *Msr1*^+/-^, and *Msr1*^-/-^ littermates. RML6-inoculated mice were checked and monitored every other day for scrapie symptoms. Mice were euthanized and brains were collected when they showed severe scrapie sign and reached the terminal stage of disease. Incubation times were calculated as the time from initial prion inoculation until terminal disease stage. We observed that all *Msr1*^+/+^, *Msr1*^+/-^, and *Msr1*^-/-^ mice succumbed to prion disease at a similar progression rate (median survival: 179 dpi for *Msr1*^+/+^ mice (*n* = 17), 186 dpi for *Msr1*^+/-^(*n* = 25) and 183.5 dpi for *Msr1*^-/-^ mice (*n* = 14), *p* = 0.99) (Fig. 2a). The above results indicate that Msr1 ablation does not overtly influence progression of prion disease.

We then analyzed and compared the histology of brain sections collected and prepared from RML6-inoculated terminally sick *Msr1*^+/+^, *Msr1*^+/-^ and *Msr1*^-/-^ mice. The classical histological characteristics of prion disease, especially the spongiform changes (or vacuolation), were observed in all mice in different groups. Lesion pattern analysis also failed to show any qualitative distinctions between different genotypes (Figure 2B). These results suggest that lack of Msr1 does not obviously affect prion-caused lesion profile in mouse brains.

If Msr1 contributed to prion clearance as it does to Aβ, *Msr1*^-/-^ mice would accumulate more PrP^Sc^ deposits in their brains. We therefore first performed PrP^Sc^ staining on brain sections prepared from RML6-inoculated terminally sick *Msr1*^+/+^, *Msr1*^+/-^ and *Msr1*^-/-^ mice. Unexpectedly, we observed a similar PrP^Sc^ deposition level and pattern in mouse brains with the different genotypes (Fig. 2b). We next performed Western blot to detect and assess proteinase K (PK)-resistant PrP^Sc^ levels in brains of terminally sick mice. We again found a similar PrP^Sc^ accumulation level in *Msr1*^+/+^, *Msr1*^+/-^, and *Msr1*^-/-^ mouse brains (Figs. 2c–d, uncropped Western blots are shown in Supplementary Fig. 3c). Additionally, we measured the prion seeding activity in brain homogenates of RML6-inoculated terminally sick *Msr1*^+/+^, *Msr1*^+/-^, and *Msr1*^-/-^ mice by real time-quaking induced conversion assay (RT-QuIC). We detected an undistinguishable 50% seeding dose (SD50) of prion in all three groups (Fig. 2e). Taken together, these results indicate that Msr1 does not play a major role in prion clearance or PrP^Sc^ accumulation in mouse brains.

### Prion-induced neuroinflammation is not affected by Msr1 deficiency

**Fig. 3:**
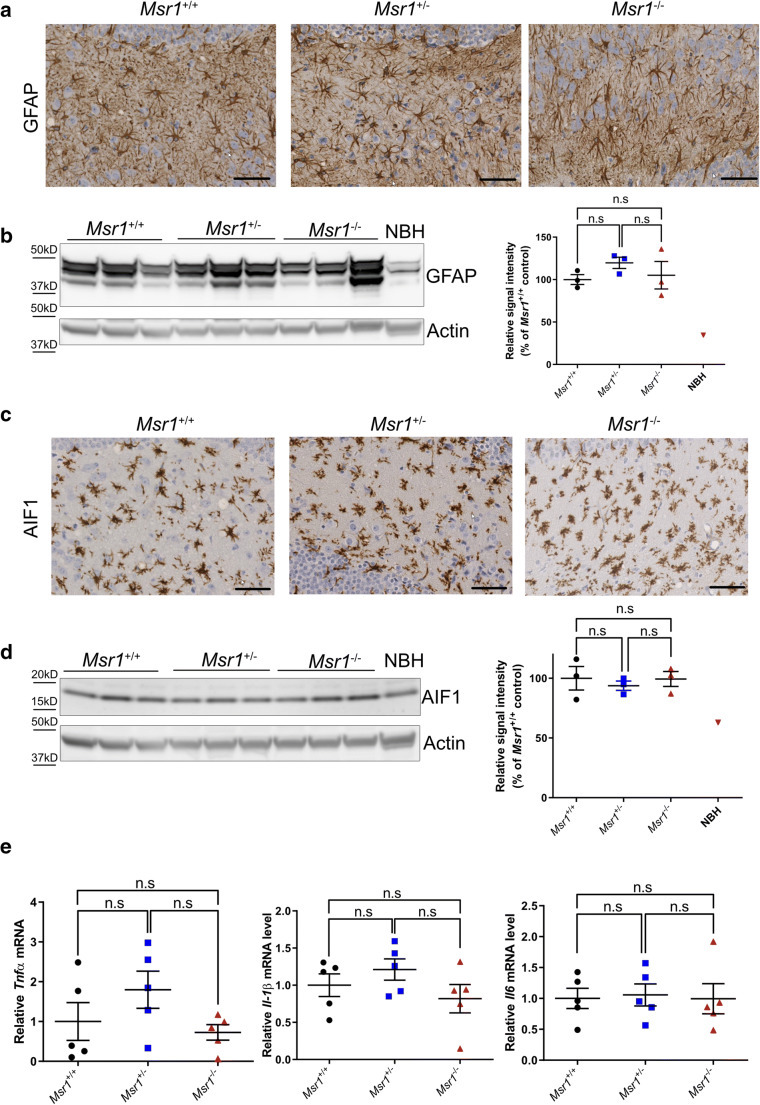
**a** Representative immunohistochemical staining for GFAP in the hippocampus of terminally sick mouse brains from *Msr1*^+/+^ (WT), *Msr1*^+/-^, and *Msr1*^-/-^ littermates. **b** Left: Western blot for GFAP in terminally sick mouse brains. Right: densitometric quantification of the Western blot showed no significant difference of GFAP levels between *Msr1*^+/+^ (WT), *Msr1*^+/-^ and *Msr1*^-/-^ littermates (*n* =3–4, n.s *p* > 0.05). **c** Representative immunohistochemical staining for AIF1 in the hippocampus of terminally sick mouse brains from *Msr1*^+/+^ (WT), *Msr1*^+/-^ and *Msr1*^-/-^ littermates. **d** Left: Western blot for AIF1 in terminally sick mouse brains. Right: densitometric quantification of the Western blot showed no significant difference of AIF1 levels between *Msr1*^+/+^ (WT), *Msr1*^+/-^, and *Msr1*^-/-^ littermates (*n* =3–4, n.s *p* > 0.05). **e** qRT-PCR of cytokines TNFα, IL-1β and IL-6 expression revealed similar mRNA levels of these cytokines (*Tnfα*, *Il1β*,*Il6*) in terminally sick *Msr1*^+/+^ (WT), *Msr1*^+/-^, and *Msr1*^-/-^ littermates (*n* = 5, n.s *p* > 0.05). Abbreviations: Msr1, macrophage scavenger receptor 1; GFAP, glial fibrillary acidic protein; AIF1, Ionized calcium binding adaptor molecule 1; WT, wild type; qRT-PCR, quantitative real-time polymerase chain reaction

Msr1 is a scavenger receptor that could modulate immune response in CNS [34–36]. To test whether Msr1 exhibits an anti-inflammatory function in prion pathogenesis, we analyzed and compared astrogliosis and microglial activation in prion-infected terminally sick *Msr1*^+/+^, *Msr1*^+/-^ and *Msr1*^-/-^ mice. First, histology and Western blots did not identify obvious differences in GFAP (glial fibrillary acidic protein, an astrocytic marker) level between the three groups with different genotypes (Figs. 3a–b, uncropped Western blots are shown in Supplementary Figs. 3d–e). We next performed histological examinations and Western blots to detect AIF1 (allograft inflammatory factor-1, a microglial marker) in RML6-inoculated terminally sick *Msr1*^+/+^, *Msr1*^+/-^, and *Msr1*^-/-^ mouse brains. We again observed similar levels of AIF1 between the three groups (Figs. 3c–d, uncropped Western blots are shown in Supplementary Figs. 3f–g). These results together suggest that Msr1 deficiency does not significantly affect prion-induced astrogliosis or microglial activation.

We next performed qRT-PCR to analyze the expression levels of proinflammatory cytokines (TNFα, IL-1β and IL6) in RML6-inoculated terminally sick *Msr1*^+/+^, *Msr1*^+/-^, and *Msr1*^-/-^ mouse brains. Again, this experiment failed to detect any overt differences in cytokine expression levels between the three groups (Fig. 3E), suggesting that Msr1 ablation does not change the cytokine profiles of prion-infected mouse brains.

## Discussion

It is becoming increasingly clear that neuroinflammation, characterized by astrogliosis and microglial activation, is a hallmark of many neurodegenerative diseases including prion diseases [37]. Emerging evidence from multiple genome-wide association studies (GWAS) suggests that microglia may play a central role in neurodegenerative diseases because most identified risk factors are expressed by microglia [11, 12, 38–42]. The molecular mechanisms by which microglia contributes to the pathogenesis of neurodegenerative diseases are the subject of intense investigations. Insight into these questions will enable not only the understanding of disease pathogenesis but also the development of novel therapeutic strategies combating these disorders [43].

As the primary tissue-resident macrophages in the CNS, microglia exert diverse functions in the brain. Microglia play important roles during brain development by pruning neuronal synapses [44]. In adult brain, microglia could modulate learning and memory [45] and constantly survey their local milieu for signals of danger and injury, thus serving as important sensors and defenders upon challenges [46, 47]. Under pathological conditions such as in prion disease, microglia are found to play an overall neuroprotective role by clearing prions. Depletion or deficiency of microglia results in impaired prion clearance, enhanced PrP^Sc^ deposition, and deteriorated prion pathogenesis [5]. These findings collectively highlight the need to better understand the molecular mechanisms underlying microglia-mediated prion clearance [3].

Msr1, a type II transmembrane glycoprotein expressed mainly by microglia in the brain [16], is a member of scavenger receptors that mediate endocytosis of a wide range of ligands including low-density lipoproteins, bacterial pathogens and dead cells [48, 49]. The role of Msr1 in neurodegeneration such as Alzheimer’s disease (AD) was first highlighted in an *in vitro* study showing that it could facilitate adhesion of microglia to fibrillar Aβ [18]. This finding was further validated by a short hairpin RNA (shRNA) library screen [21]. In mouse models of AD, Msr1 deficiency impairs clearance of soluble Aβ and results in increased Aβ deposition and early mortality, whereas pharmacological upregulation of Msr1 leads to enhanced Aβ clearance [21, 22]. Therefore, it is reasonable to speculate that Msr1 could play a scavenger function for a broad range of misfolded protein aggregates including prions.

In this study, we tested whether Msr1 plays a scavenger function in prion pathogenesis, similar to that in mouse models of AD. Firstly, in contrast to what was observed in aged AD mouse models [50, 51], Msr1 expression was found to be upregulated at the terminal stage upon prion infection. After prion inoculation, we observed that *Msr1*^-/-^ mice experienced a similar incubation time compared with *Msr1*^+/-^ and wild-type littermates, suggesting that Msr1 deficiency does not affect prion disease progression. Notably, the PrP^Sc^ level and the prion seeding activity were not detectably affected by Msr1 deficiency, indicating that Msr1 is not a major contributor to prion clearance. Moreover, prion-induced neuroinflammation including astrocytosis and microglial activation was not altered in Msr1^-/-^ mouse brains, suggesting that Msr1 deficiency does not overtly affect prion-induced neuroinflammation. Together, these results indicate that Msr1 functions differently in AD and prion diseases.

### Limitations of the current study

In the present study, we found that *Msr1* expression was upregulated in terminally sick prion-infected mouse brains detected by both qRT-PCR and RNA-seq. Longitudinal study showed that upregulation of Msr1 was not merely due to increased microglia number. Although both analyses were based on brain tissue containing a mixture of various cell types, a cell-type specific ribosomal profiling again found that Msr1 was significantly upregulated in microglia only at the terminal stage. Orthogonal methodologies, such as cell sorting followed by RNA-seq, would be required to confirm the microglia-specific *Msr1* expression pattern along the prion progression. Nevertheless, the late upregulation of Msr1 may explain that Msr1 have little impact on the prion pathogenesis. The varied temporal pattern and magnitude of changes of Msr1 expression between prion disease and AD may explain its differential functions in these diseases.

We performed prion infection experiments with an appropriate number of animals (n=14~25 mice per group) and conclude, with a high degree of confidence, that Msr1 deficiency did not overtly affect progression of prion disease. We randomly divided the samples into subgroups for either biochemical analysis or histology encompassing a relatively small number of samples (*n* = 3–5 mice per group). Because of the increased stochasticity inherent to such subgroups, we observed some variations in these analyses. Notwithstanding these limitations, our results point towards the same direction and are congruent with our conclusion that Msr1 does not play a major role in prion pathogenesis.

## Conclusion

Collectively, these results indicate that Msr1 does not play a major role in prion pathogenesis. Together with the discrepant observations of TREM2 in AD and prion disease[9, 14], this study suggests that microglia-mediated phagocytosis and clearance of Aβ and prion may adopt distinct molecular pathways. Further studies are needed to investigate molecular mechanisms underlying microglial uptake and clearance of prions.

## Supplementary Information

Supplemental Figure 1 Western blots using commercial anti-Msr1 antibodies that could not detect specific Msr1 band on brains collected from wild type mice. *Msr1*^-/-^ mouse brains were used as negative control. (PNG 512 kb)

High resolution image (TIFF 734 kb)

Supplemental Figure 2 RNA-Seq data of *Msr1* (A) and microglia markers (*Aif1*, *Cd68* and *Itgax*) (B-D) expression in hippocampi at different time points after prion inoculation. (n=3, except n=2 at 20wpi for NBH, *P<0.05; ***P<0.001). (E) Ribosomal profiling of *Msr1* expression in CX3CR1-positive microglia (n=3, except n=2 at terminal stage, ****P<0.0001). Abbreviations: *Msr1*, macrophage scavenger receptor 1 gene; *Aif1*, Ionized calcium binding adaptor molecule 1 gene; *Cd68*, cluster of differentiation 68 gene; *Itgax*, integrin subunit alpha x gene; RML6, Rocky Mountain Laboratories scrapie strain, passage 6; NBH, noninfectious brain homogenates. (PNG 451 kb)

High resolution image (TIFF 737 kb)

Supplemental Figure 3 Full images of the cropped Western blots in Figure 1C (A-B), 2C (C), 3B (D-E) and 3D (F-G). (PNG 802 kb)

High resolution image (TIFF 1216 kb)

## Data Availability

The authors confirm that the data and material supporting the findings of this study are available within the article and its supplementary materials.
