# Supplementary figures and images for: The role of macrophage scavenger receptor 1 (Msr1) in prion pathogenesis
Source: J Mol Med (Berl). 2021 Mar 23;99(6):877–87. doi: 10.1007/s00109-021-02061-7 (PMC8164582; doi:10.1007/s00109-021-02061-7)

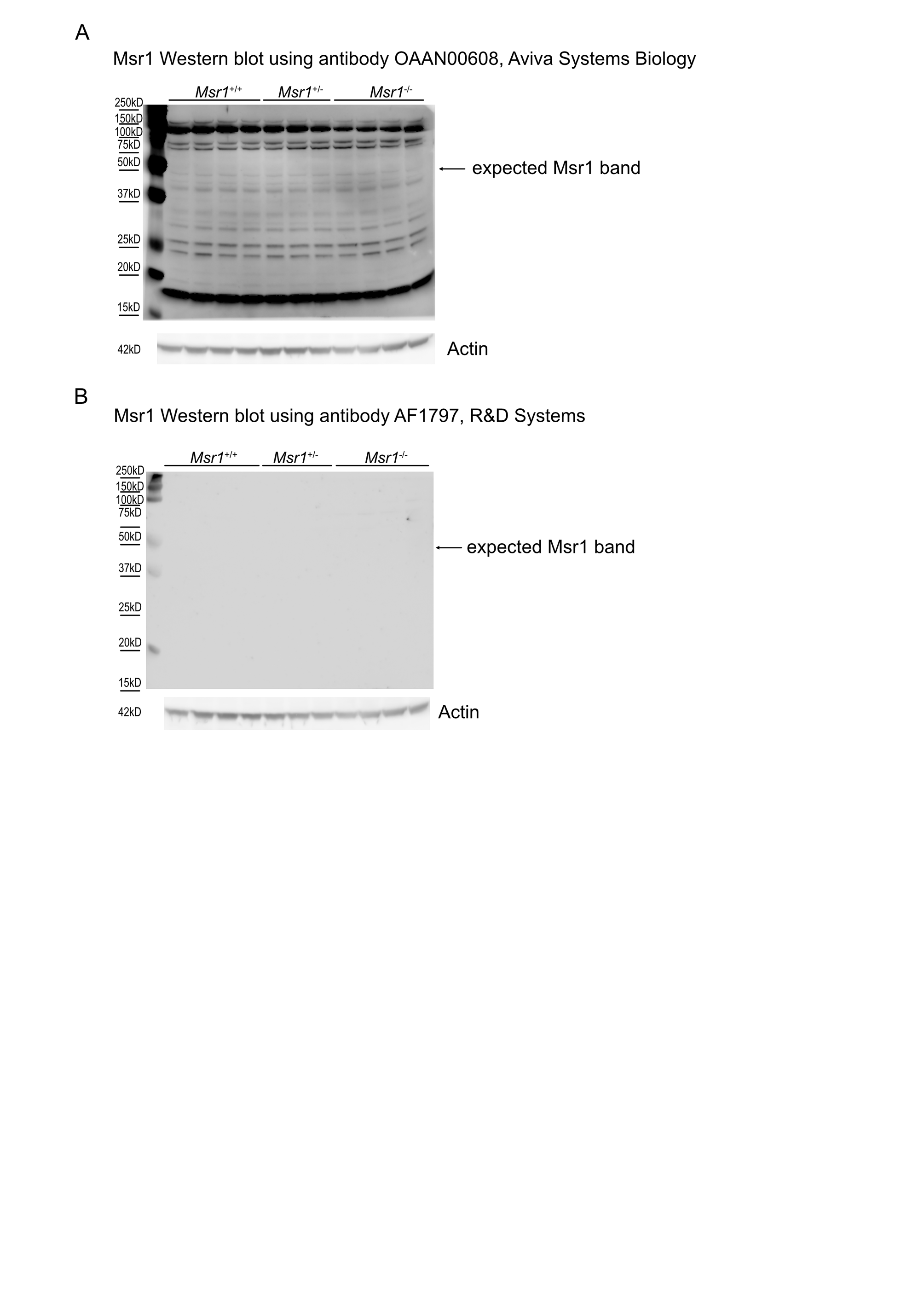

Supplement: Supplementary file 1 — Western blots using commercial anti-Msr1 antibodies that could not detect specific Msr1 band on brains collected from wild type mice. Msr1-/- mouse brains were used as negative control. (PNG 512 kb) [file 109_2021_2061_Fig4_ESM.png]

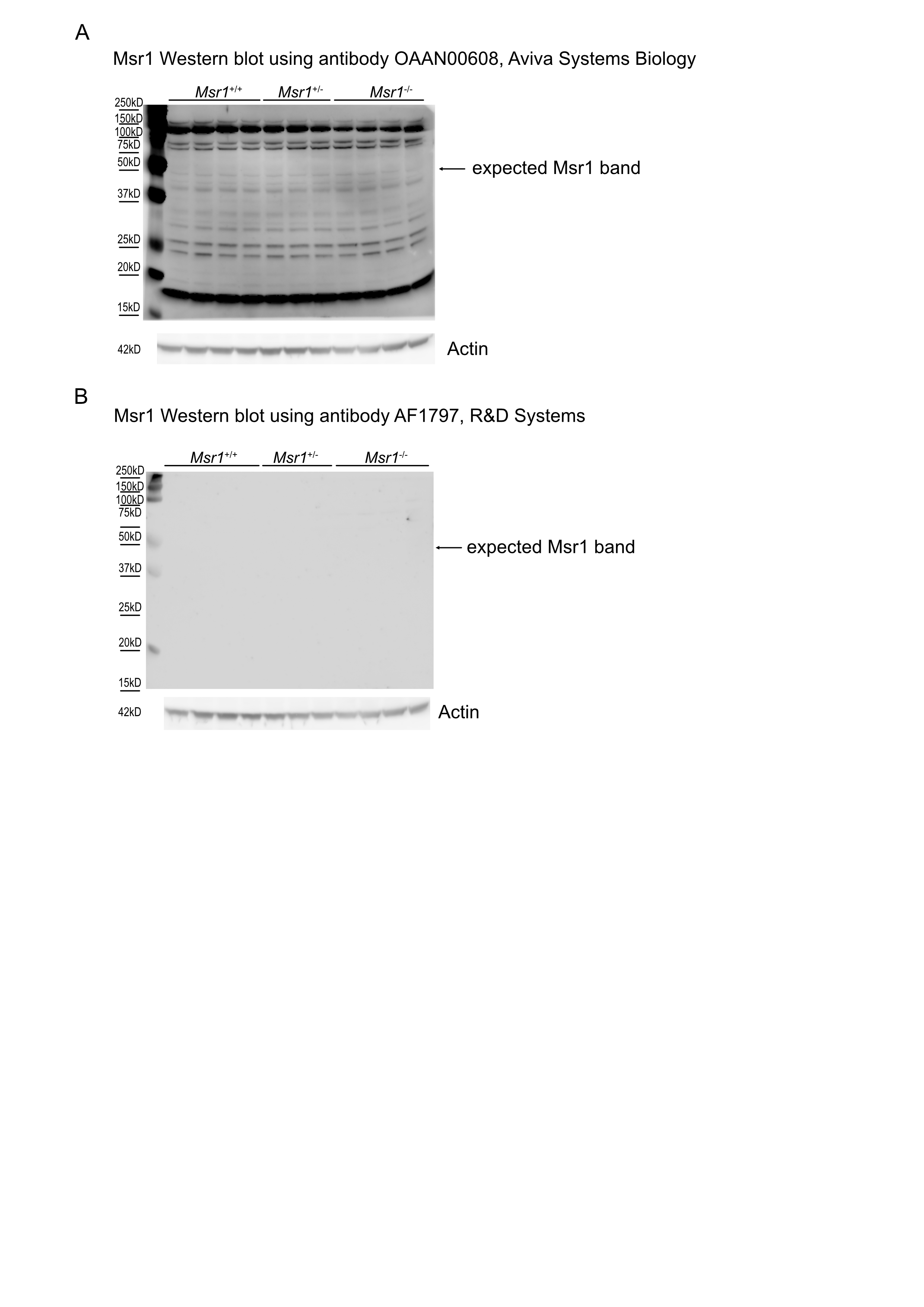

Supplement: Supplementary file 2 — High resolution image (TIFF 734 kb) [file 109_2021_2061_MOESM1_ESM.tiff]

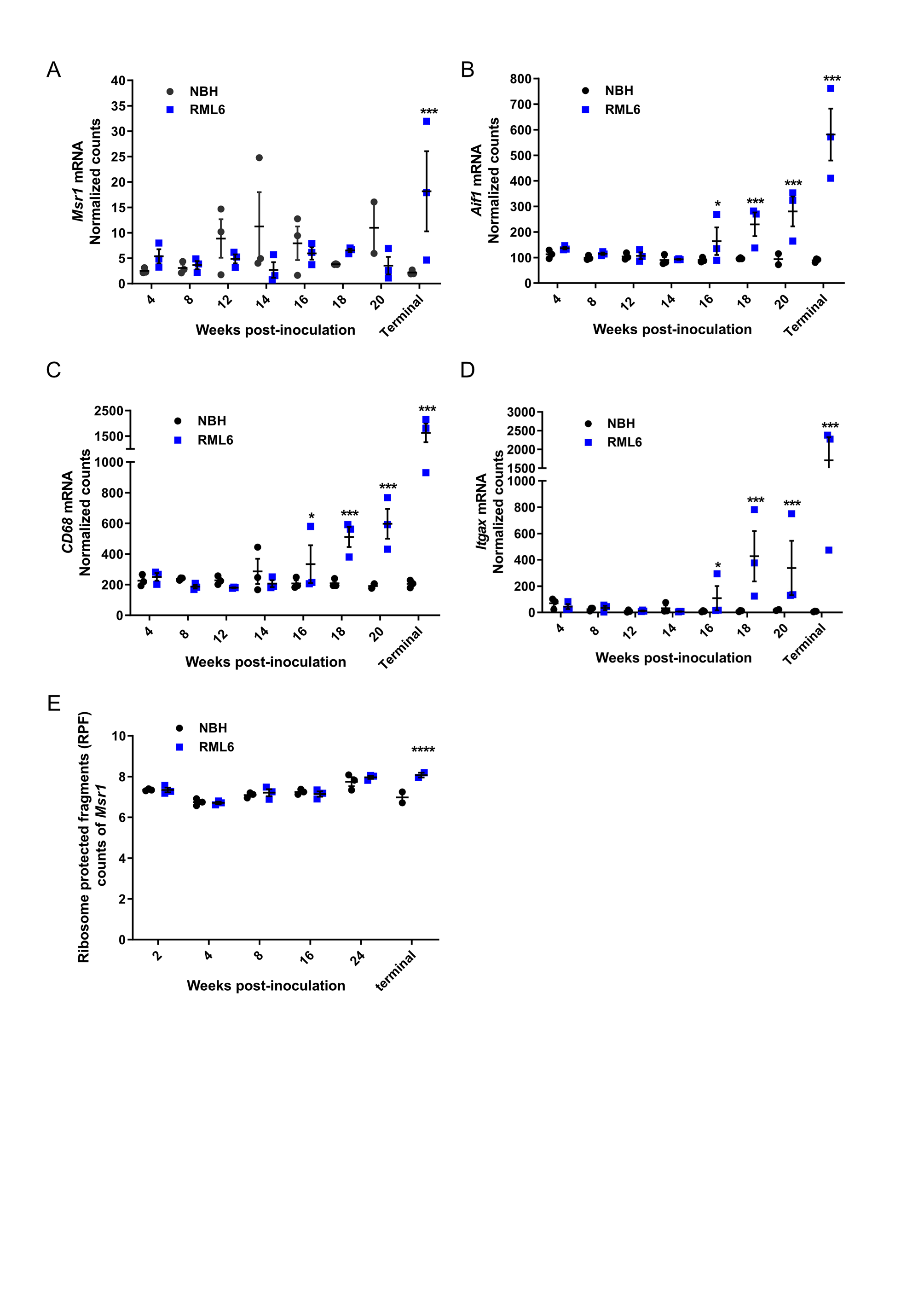

Supplement: Supplementary file 3 — RNA-Seq data of Msr1 (A) and microglia markers (Aif1, Cd68 and Itgax) (B-D) expression in hippocampi at different time points after prion inoculation. (n=3, except n=2 at 20wpi for NBH, *P<0.05; ***P<0.001). (E) Ribosomal profiling of Msr1 expression in CX3CR1-positive microglia (n=3, except n=2 at terminal stage, ****P<0.0001). Abbreviations: Msr1, macrophage scavenger receptor 1 gene; Aif1, Ionized calcium binding adaptor molecule 1 gene; Cd68, cluster of differentiation 68 gene; Itgax, integrin subunit alpha x gene; RML6, Rocky Mountain Laboratories scrapie strain, passage 6; NBH, noninfectious brain homogenates. (PNG 451 kb) [file 109_2021_2061_Fig5_ESM.png]

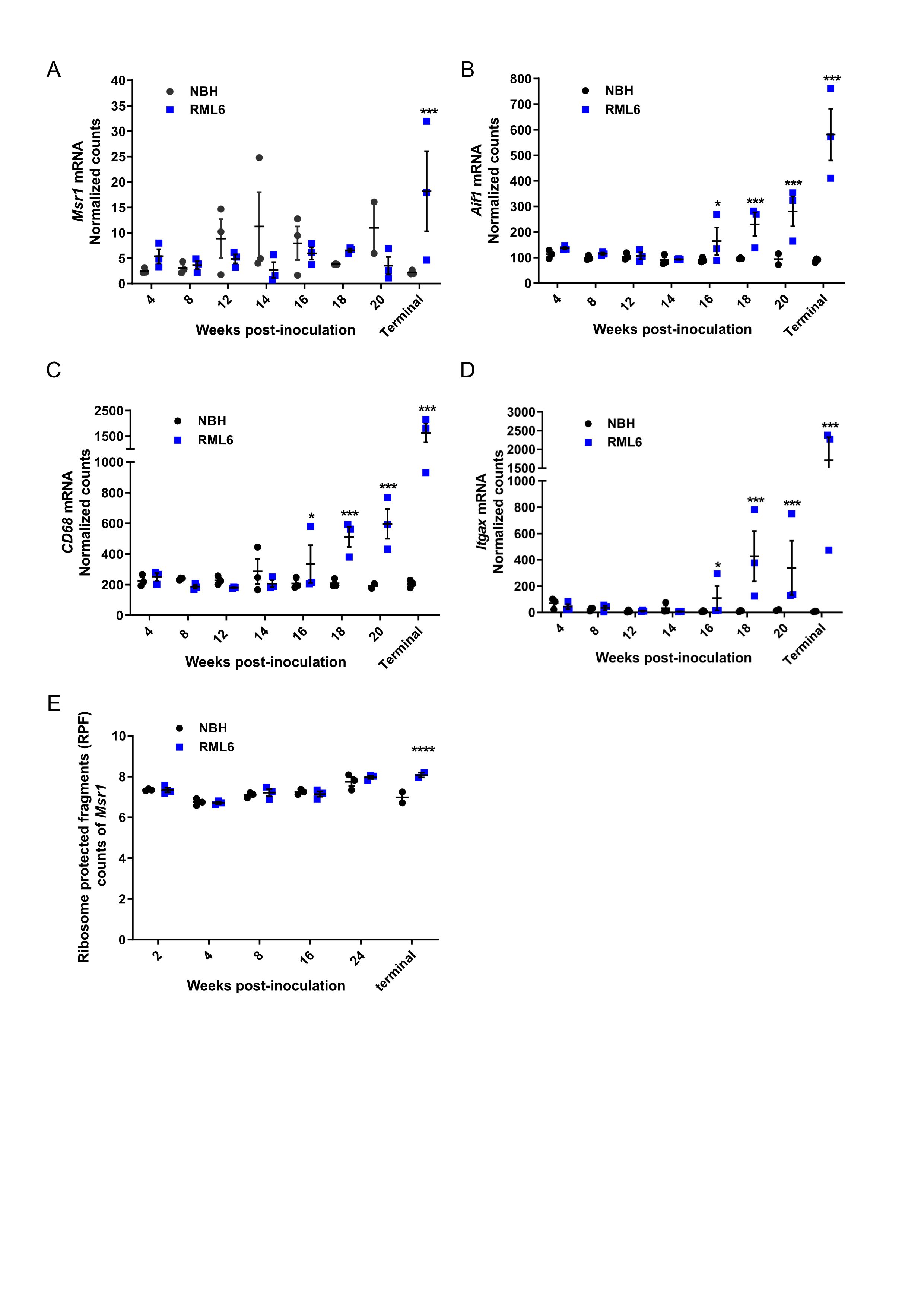

Supplement: Supplementary file 4 — High resolution image (TIFF 737 kb) [file 109_2021_2061_MOESM2_ESM.tiff]

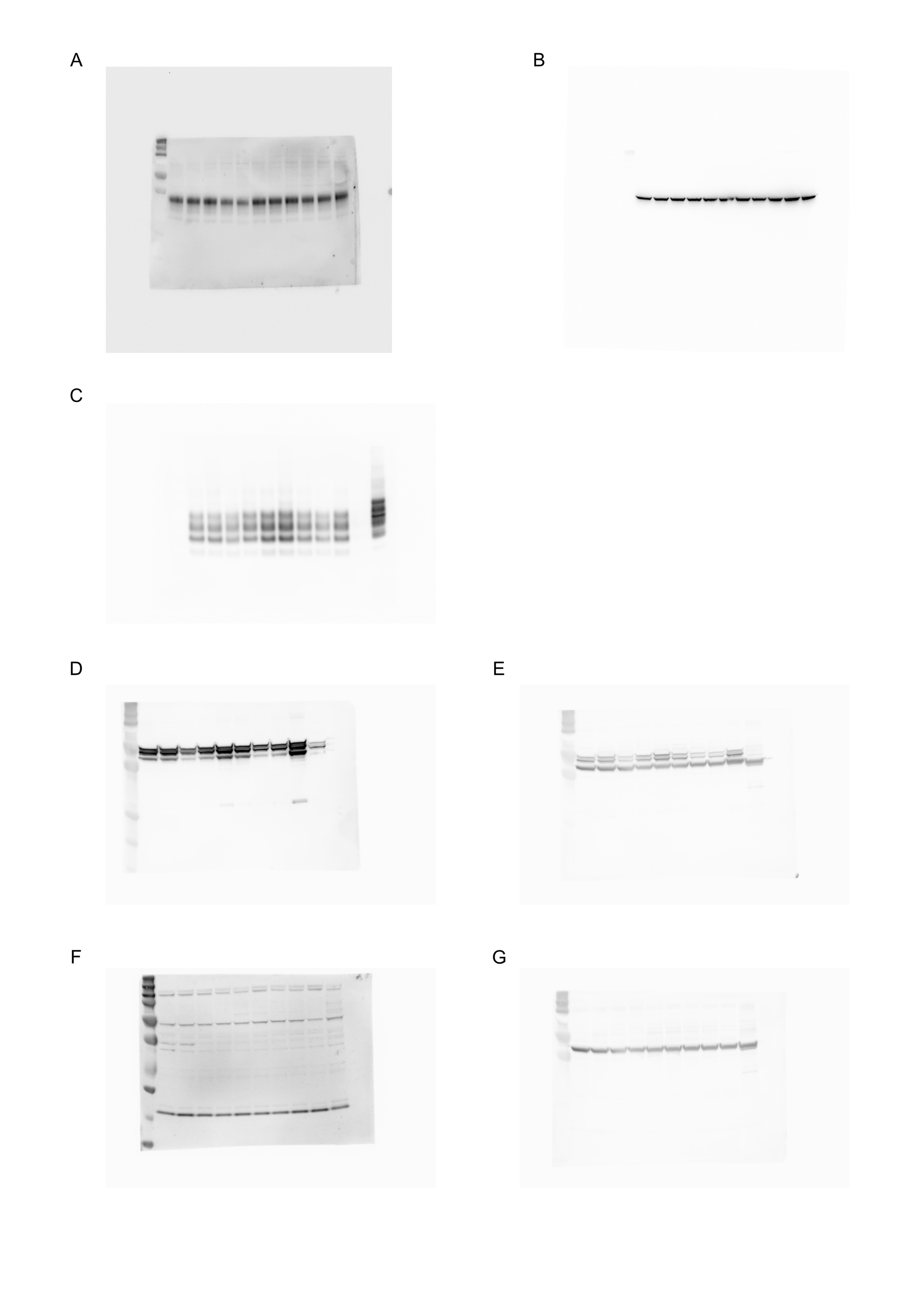

Supplement: Supplementary file 5 — Full images of the cropped Western blots in Figure 1C (A-B), 2C (C), 3B (D-E) and 3D (F-G). (PNG 802 kb) [file 109_2021_2061_Fig6_ESM.png]

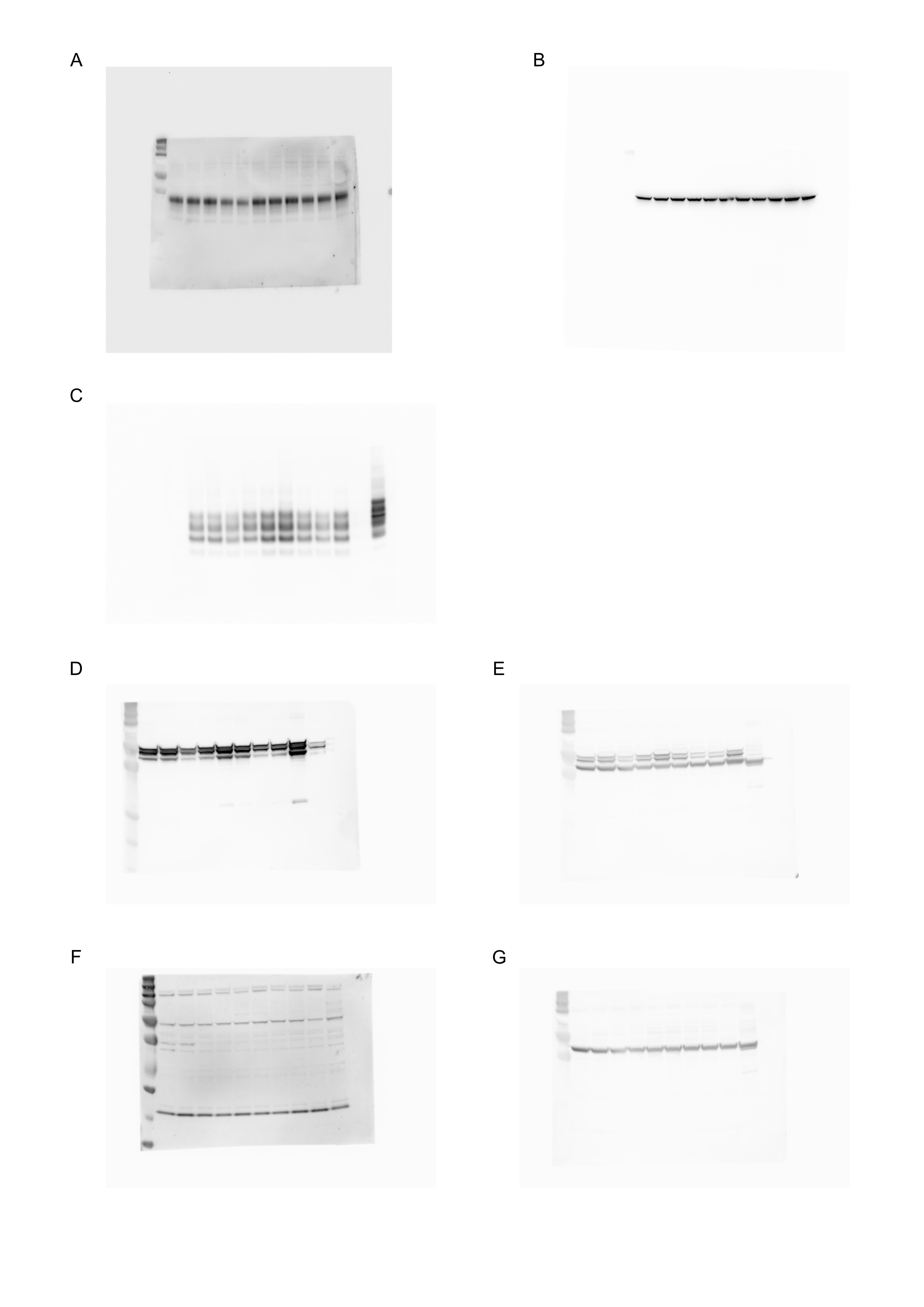

Supplement: Supplementary file 6 — High resolution image (TIFF 1216 kb) [file 109_2021_2061_MOESM3_ESM.tiff]
